# Asymptomatic transmission and the resurgence of *Bordetella pertussis*

**DOI:** 10.1186/s12916-015-0382-8

**Published:** 2015-06-24

**Authors:** Benjamin M. Althouse, Samuel V. Scarpino

**Affiliations:** Santa Fe Institute, Santa Fe, NM USA

**Keywords:** *Bordetella pertussis*, Whooping cough, Asymptomatic infection, Phylodynamic analysis, Stochastic disease dynamical modeling, Vaccination policy

## Abstract

**Background:**

The recent increase in whooping cough incidence (primarily caused by *Bordetella pertussis*) presents a challenge to both public health practitioners and scientists trying to understand the mechanisms behind its resurgence. Three main hypotheses have been proposed to explain the resurgence: 1) waning of protective immunity from vaccination or natural infection over time, 2) evolution of *B. pertussis* to escape protective immunity, and 3) low vaccine coverage. Recent studies have suggested a fourth mechanism: asymptomatic transmission from individuals vaccinated with the currently used acellular *B. pertussis* vaccines.

**Methods:**

Using wavelet analyses of *B. pertussis* incidence in the United States (US) and United Kingdom (UK) and a phylodynamic analysis of 36 clinical *B. pertussis* isolates from the US, we find evidence in support of asymptomatic transmission of *B. pertussis*. Next, we examine the clinical, public health, and epidemiological consequences of asymptomatic *B. pertussis* transmission using a mathematical model.

**Results:**

We find that: 1) the timing of changes in age-specific attack rates observed in the US and UK are consistent with asymptomatic transmission; 2) the phylodynamic analysis of the US sequences indicates more genetic diversity in the overall bacterial population than would be suggested by the observed number of infections, a pattern expected with asymptomatic transmission; 3) asymptomatic infections can bias assessments of vaccine efficacy based on observations of *B. pertussis*-free weeks; 4) asymptomatic transmission can account for the observed increase in *B. pertussis* incidence; and 5) vaccinating individuals in close contact with infants too young to receive the vaccine (“cocooning” unvaccinated children) may be ineffective.

**Conclusions:**

Although a clear role for the previously suggested mechanisms still exists, asymptomatic transmission is the most parsimonious explanation for many of the observations surrounding the resurgence of *B. pertussis* in the US and UK. These results have important implications for *B. pertussis* vaccination policy and present a complicated scenario for achieving herd immunity and *B. pertussis* eradication.

**Electronic supplementary material:**

The online version of this article (doi:10.1186/s12916-015-0382-8) contains supplementary material, which is available to authorized users.

## Background

**Fig. 1:**
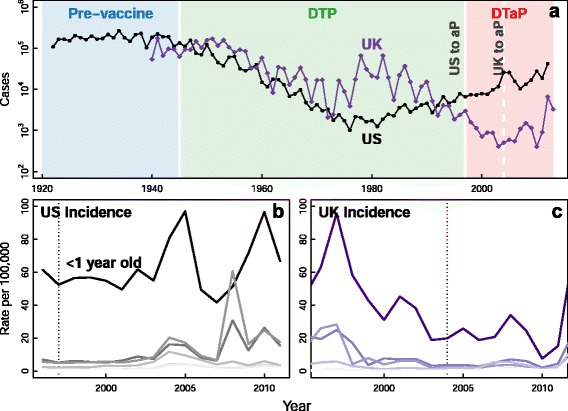
Increase in *B. pertussis* incidence over time. Panel **a** shows *B. pertussis* cases in the United States from 1922 through 2012 and in the United Kingdom from 1940 through 2013 (from: [17] and [70]). Shaded regions correspond to the pre-vaccine era, the DTP era, and the DTaP era, respectively. Panels **b** and **c** show the incidence of *B. pertussis* by age group with darker color indicating younger ages in the US and UK, respectively. Infants less than 1 year old are labeled in darkest colors

**Fig. 2:**
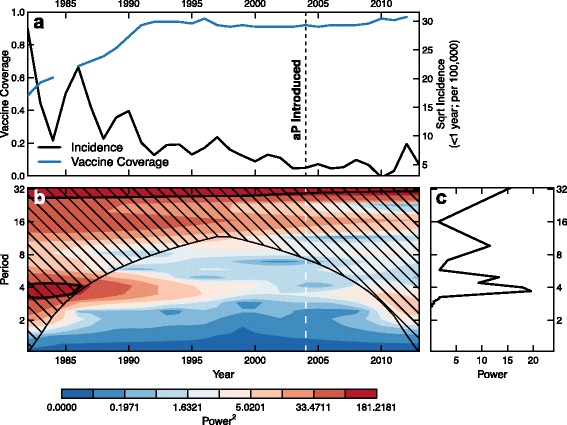
Disruption of *B. pertussis* cycles by vaccination. Panel **a** shows the square root of *B. pertussis* cases in infants less than 1 year old in the United Kingdom from 1982 through 2013 (in black) and the percentage vaccine coverage over the same period (in blue). Vertical dashed line indicates the switch to an aP vaccination schedule. Panel **b** shows the standard wavelet spectrum of the incidence in panel **a**; panel **c** shows the Fourier spectrum of the incidence. As vaccination coverage increases (1985 through about 1991) we see a decrease in the power of cycles of approximately 4 years. We begin to see an increase in this power after the introduction of aP vaccination in 2004, suggesting transmission patterns similar to those observed in the pre-transmission-blocking vaccine era

Many countries have seen a startling increase in the incidence of *Bordetella pertussis*, an important causative agent of whooping cough, over the past 20 years [1]. In the United States (US), 2012 saw more diagnosed *B. pertussis* cases than in any year since 1955 (Fig. 1 and [2], accessed 20 January 2015). The United Kingdom (UK) has seen a similarly startling rise, with more cases occurring in 2013 than since the vaccine refusal era of the 1970s and 1980s (data available here: [3], accessed 20 January 2015). Two general hypotheses have been proposed to explain the rise in *B. pertussis* incidence: either vaccination coverage is too low, where individuals remain unvaccinated or unvaccinated susceptible individuals move into populations; or vaccinated individuals can still become infected [1, 4]. While vaccination coverage has likely played a role in increasing incidence, coverage has historically been high [1, 5], raising the likelihood that the resurgence is — at least in part — due to low vaccine effectiveness [6].

To date, three primary mechanisms have been proposed to explain why vaccinated individuals can become infected with *B. pertussis*: 1) the vaccine mounted a sterilizing immune response that waned over time [7], 2) the pathogen evolved to escape sterilizing immunity induced by the vaccine [8], or 3) the vaccine failed to induce sterilizing immunity to the pathogen [9]. While the first two mechanisms have received considerable attention, the third was only recently proposed by Warfel, Zimmerman, and Merkel (2014) [9]. In their study, Warfel et al. used non-human primates as a model for *B. pertussis* infection, and found evidence that individuals vaccinated with current acellular *B. pertussis* vaccines (aP) can become asymptomatically infected, and can then transmit infection to susceptible individuals. The potential for this type of vaccine failure has been observed in humans where reanalyses of aP vaccine studies revealed that individuals vaccinated with components of the aP vaccine were protected against disease, but not bacterial colonization [10, 11]. This is in addition to the extant, but limited, evidence for natural asymptomatic infection [12–14].

Warfel et al. point out that asymptomatic infection in aP vaccinated individuals, and subsequent transmission, may partially account for the increase in observed *B. pertussis* incidence. However, from a public health perspective, the presence of vaccine-induced or naturally infected asymptomatic individuals who transmit disease could have consequences beyond facilitating an increase in incidence. In response to Warfel et al., Domenech de Cellès et al. (2014) [15] argue that a reduction in incidence among unvaccinated individuals in a population with high aP coverage shows that aP must reduce *B. pertussis* transmission to some extent. It may be that aP vaccinated infected people are less efficient at transmitting *B. pertussis* compared with unvaccinated infected people, though it is not clear to what extent [16].

Here, we examine incidence and genetic data to provide empirical support for asymptomatic transmission and then construct mathematical models of *B. pertussis* transmission to explore the public health consequences of asymptomatic transmission. Our results suggest that: 1) there is strong empirical support for asymptomatic transmission from both the epidemiological and genomic data; 2) the presence of asymptomatic transmitters will bias estimates of vaccine efficacy derived from observations of stochastic fadeouts across cities; and 3) asymptomatic transmission provides the most parsimonious explanation for many of the observed patterns associated with current *B. pertussis* dynamics in the US and UK (that is, the resurgence of cases, the changes in age-specific attack rates, the observed level of bacterial genetic variation, and the failure of ring-vaccinating, or “cocooning”, unvaccinated infants). The results on vaccination have important public health and clinical implications, especially related to recommendations for isolating unvaccinated or partially vaccinated infants.

## Methods

### Empirical data

All reported *B. pertussis* cases in the United States from 1922 through 2012 were obtained from the US Centers for Disease Control (CDC) [17], and all reported *B. pertussis* cases for the United Kingdom from 1940 through 2013 were obtained from Public Health England (PHE, data available here: [18], accessed 8 October 2014). Age-specific *B. pertussis* incidence was obtained from the CDC and the PHE. Population sizes for the denominators were obtained from the US Census through the CDC, and for the UK from the UK Office for National Statistics (data available here: [19], accessed 8 October 2014). Historic US incidence data come from the CDC’s Public Health Reports and Morbidity and Mortality Weekly Reports (MMWR), digitized and downloaded from Project Tycho [20, 21].

### The model

We formulate deterministic and stochastic Susceptible, Infected, Removed (SIR) models of *B. pertussis* transmission [22–24]. Briefly, susceptible individuals are born at rate *μ*, where they are vaccinated with either the whole-cell (wP) or acellular (aP) *B. pertussis* vaccine, depending on which vaccine is currently in use. Our model includes three vaccine epochs: one without vaccination, one with only wP vaccination, and one with only aP vaccination. These epochs are non-overlapping, similar to the advent of wP and its replacement by aP [25]. We assume those vaccinated with wP are completely immune to infection (see Discussion). Those vaccinated with aP move into a vaccinated class where they can become asymptomatically infected (that is, they incur a direct benefit from vaccination because they will not develop symptomatic disease [26, 27]). Unvaccinated individuals become infected with *B. pertussis* at rate *β* and become symptomatic with probability *σ* (sensitivity to which is explored in the supplementary information Additional file 1), and aP vaccinated individuals become asymptomatically infected at rate *β*. We assume no difference in transmissibility between symptomatic and asymptomatic individuals (see Discussion and Additional file 1). Individuals recover from symptomatic and asymptomatic infection at rates *γ*_*s*_ and *γ*_*a*_. Individuals die at rate *ν*, which we set equal to *μ* to keep the population size constant. Individuals can wane from protective immunity at rate *ω*. The equations governing transmission dynamics are:

(1) $$\begin{array}{@{}rcl@{}} {}S'(t) &=& \mu \cdot (1-wP-aP)-\beta [I_{s}(t)+I_{a}(t)] S(t) {}\\ &&+ \omega R(t) -\nu S(t)  \end{array} $$

(2) $$\begin{array}{@{}rcl@{}} {}I_{s}'(t) &=& \beta \sigma [I_{s}(t)+I_{a}(t)] S(t)-\gamma_{s} I_{s}(t) - \nu I_{s}(t) \end{array} $$

(3) $$\begin{array}{@{}rcl@{}} {}I_{a}'(t) &=& \beta (1-\sigma) [I_{s}(t)+I_{a}(t)] S(t)+\beta [I_{s}(t)+I_{a}(t)] \\&&V(t)-\gamma_{a} I_{a}(t) - \nu I_{a}(t) \end{array} $$

(4) $$\begin{array}{@{}rcl@{}} {}V'(t) &=& \mu \cdot aP-\beta [I_{s}(t)+I_{a}(t)] V(t)- \nu V(t) \end{array} $$

(5) $$\begin{array}{@{}rcl@{}} {}R'(t) &=& \mu \cdot wP +\gamma_{s} I_{s}(t)+\gamma_{a} I_{a}(t) - \omega R(t) - \nu R(t)  \end{array} $$

We assume that the aP vaccine has 100 % efficacy in preventing disease; however, this is a conservative assumption with respect to our conclusions. We formulate both deterministic and stochastic (Gillespie stochastic simulation algorithm [28] with the binomial tau-leap approximation [29]) versions of the model. Analytical expressions for the basic reproduction number (*R*_0_) and model equilibria are given in Additional file 1.

### Phylodynamic analysis

We fit a series of phylodynamic models to concatenated single nucleotide polymorphisms (SNPs) identified from whole *B. pertussis* genome sequences isolated from patients infected in the US between 1935 and 2005 [30]. The resulting data set contained 36 isolates: 2 from the pre-vaccine era, 8 from the wP vaccine era, and 26 from the aP vaccine era, each with a nucleotide length of 5,414 bases. Sequencing, alignment, and variant calling were all performed by Bart et al. (2014) [30].

Using this data set, we inferred the parameters of a phylodynamic model using Bayesian Markov chain Monte Carlo (MCMC) methods implemented in BEAST 2.1 [31] with a Hasegawa-Kishino-Yano (HKY) substitution model, as suggested by jModelTest 2.1 [32], and a birth-death skyline tree model with serial sampling [33]. The underlying model is a stochastic birth-death process, which is the same underlying stochastic process for models such as SI, SIR, and SEIR. We are interested in two parameters: the birth rate, which is the rate at which new cases enter the population through transmission; and the sampling rate, which is the rate at which cases leave the population because they are sampled. We make the assumption that sampling a case, that is, collecting *B. pertussis* bacteria and sequencing the genome, removes that infected individual from the population. This is an assumption, but it appears logical: once an individual is diagnosed with whooping cough, and *B. pertussis* isolated, the individual is no longer transmitting due to having cleared the infection, or being socially removed from susceptible individuals. Convergence of the model was obtained with two independent MCMC runs of more than 75 million generations and confirmed with effective sample size (ESS) values above 200 calculated with TRACER v1.6. Similar results were obtained using both a relaxed lognormal and relaxed exponential molecular clock [34].

## Results

### Empirical evidence of asymptomatic transmission

Despite its perceived importance, ascertaining evidence for asymptomatic transmission for any disease remains a challenge. This has been especially true with *B. pertussis*, where identifying individuals asymptomatically infected has historically been difficult [12–14]. Perhaps the most compelling piece of evidence for asymptomatic transmission is the documented failure of cocooning to protect newborns [35, 36], which can only be parsimoniously explained by asymptomatic transmission [9]. Other empirical patterns are expected — in both incidence and genetic data — if asymptomatic transmission is occurring as a result of the aP vaccine preventing symptomatic disease, but failing to block transmission. Here we provide empirical observations, which are both consistent with and in total most parsimoniously accounted for by asymptomatic transmission.

Figure 2 shows a wavelet analysis of *B. pertussis* incidence in the UK in unvaccinated and under-vaccinated infants (<1 year old), both considered indicators of overall *B. pertussis* transmission in a population [37]. As wP vaccine coverage increases to greater than 90 % (from the early 1980s to the early 1990s) we see a disruption of the natural approximately 4-year periodicity of *B. pertussis* cases driven by susceptible turnover [38, 39]. However, the switch from wP to aP in the UK coincides with the return of cyclic patterns, which are similar to the approximately 4-year periodicity seen in the pre-vaccine era.

**Fig. 3:**
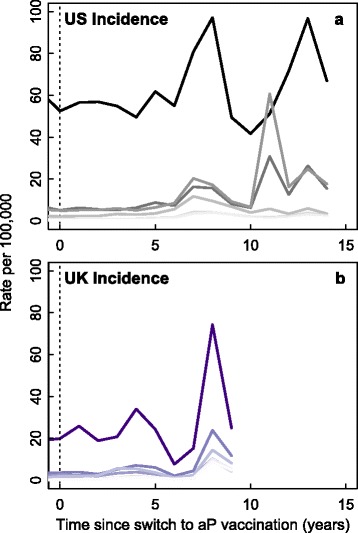
Increase in *B. pertussis* incidence after switch to aP vaccination. Figure compares the incidence of *B. pertussis* after the switch to aP vaccination in the US (panel **a**) and the UK (panel **b**). Time since the switch is presented on the x-axis. Note the similarities in the timing of spikes in incidence after the switch to aP vaccination

A second line of evidence comes from changes in age-specific attack rates in the US and UK. Both countries switched to the aP vaccine; however, the US switch occurred seven years before the UK switch. Changes in age-specific attack rates are observed in both countries after switching to the aP vaccine, and appear similar when viewed as time since the switch to aP (Fig. 3). Another important facet of these data is that within both countries the rise in attack rates is synchronous across age groups. These two observations would be an expected consequence of asymptomatic transmission from aP vaccinated individuals as fully aP vaccinated individuals become old enough to attend school and become infected. These observations are challenging to explain with either waning immunity or pathogen evolution alone; for example, waning immunity should most often induce asynchrony in age-specific attack rates [22]. Increases in incidence could be explained by heterogeneous vaccine coverage or changing vaccine effectiveness, which we are assuming in switching from the wP to aP vaccine.

Lastly, we find evidence consistent with asymptomatic transmission using the results of a transmission-oriented phylodynamic model [31] fit to 36 US *B. pertussis* genomes [30]. Asymptomatic transmission is expected to cause a mismatch between incidence estimated from case data and incidence reconstructed from genetic data. Intuitively, this happens because the population genetic variation of the bacteria is a function of the entire infected population size, while case data can only come from symptomatic individuals. This is true even if bacterial sequences are only collected from symptomatic individuals, assuming asymptomatic and symptomatic individuals are mixing with each other. The observed pattern can only be accounted for by asymptomatic transmission or underreporting. Because whooping cough reporting has increased in recent years [40, 41], asymptomatic transmission remains the only plausible explanation for the observed population genomic pattern.

**Fig. 4:**
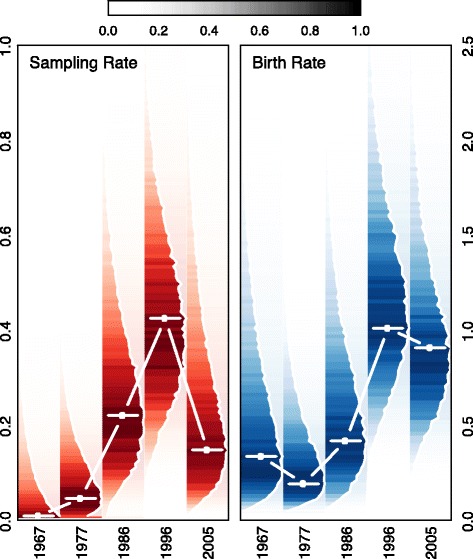
Phylodynamic analyses. Figure shows the sampling rate and birth rate derived from the BEAST analysis for the 36 US *B. pertussis* genomes. Solid white lines with square boxes indicate the posterior median, with the shaded region indicating the 95 % highest posterior density. Darker colors are associated with regions of higher posterior density, with the shape representing the actual posterior density. Despite the birth rate remaining higher after the switch to aP, the sampling rate declines. This pattern would be expected with an increasing rate of asymptomatic transmission

Figure 4 shows the changes in sampling and rates since the mid-1960s for the US. We see an increase in the birth rate after the switch to aP, as expected given the rise in incidence; however, we see a decrease in the sampling rate after the switch to aP. This mismatch between the birth rate and sampling rate indicates that, despite increasing numbers of new *B. pertussis* cases, the fraction of cases identified decreases. Importantly, the estimated decrease in the sampling rate coincides with the time-window containing the largest number of sampled bacteria and in an era with the greatest accuracy in the laboratory methods used in diagnosing *B. pertussis* cases [42]. This finding is indicative of an increase in the overall genetic diversity of *B. pertussis* in the population after the US switched from the wP to the aP vaccine. Natural selection favoring a vaccine escape variant would be expected to reduce the genetic variation and increase the estimated sampling rate, as seen in Australia by Bart et al. [30]. Additionally, waning immunity could result in an increase in the genetic diversity of the bacterial population; however, without systematic underreporting it would not result in a lower estimated sampling rate. Again, we conclude that asymptomatic transmission remains the only parsimonious explanation for the observed phylodynamic patterns.

### Consequences of asymptomatic transmission: biased estimates of vaccine effectiveness

**Fig. 5:**
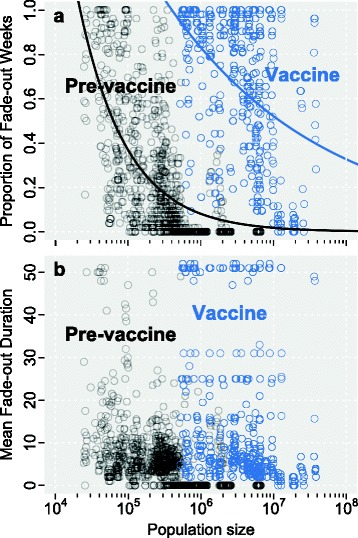
Comparing disease-free weeks in pre- and post-vaccination scenarios. Panel **a** shows the proportion of disease-free weeks (fade-outs) per year for the 50 US states in the pre-vaccine (1920–1945, black points and line) and post-vaccine (2006–2013, blue points and line) eras. Lines indicate best-fit exponential curves. Panel **b** shows the mean duration of consecutive disease-free weeks in both eras

Historically, a key indicator of success for vaccination programs is a reduction in pathogen transmission in populations, as measured by the mean proportion of disease-free weeks [24, 43, 44]. Figure 5 presents the proportion of reported *B. pertussis*-free weeks (fade-outs, panel **a**) and mean duration of fade-outs (panel **b**) for the 50 US states in pre-vaccine (1920–1945) and post-vaccine (2006–2013) epochs. The pre-vaccine epoch clearly has fewer weeks with no reported *B. pertussis* cases than does the modern vaccine epoch. This has been widely interpreted as evidence for decreasing levels of *B. pertussis* transmission and some degree of herd immunity afforded by the vaccine [7, 15, 44]. However, if situational awareness is impaired by a vaccine that protects against symptomatic disease but does not block transmission, interpreting reported *B. pertussis*-free weeks can be misleading.

**Fig. 6:**
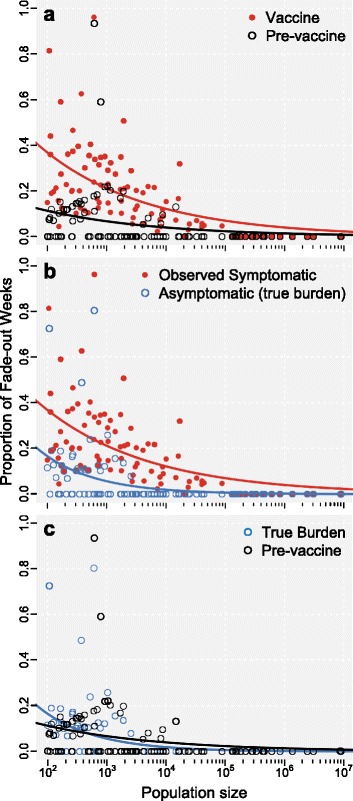
Changes in transmission in pre- and post-vaccination scenarios? Figure shows the proportion of disease-free weeks (fade-outs) for various population sizes from the stochastic formulation of the model. Panel **a** compares the symptomatic cases in the aP vaccination era with those in the pre-vaccine era; panel **b** compares the symptomatic to asymptomatic cases in the vaccine era; panel **c** compares the asymptomatic cases in the post-vaccine era with those in the pre-vaccine era. These results demonstrate no changes in transmission due to vaccination. Parameters: birth rate (*μ*) = death rate (*ν*) = 1/75 years ^−1^; recovery rates for symptomatic (*γ*_*s*_) and asymptomatic (*γ*_*a*_) = 14 days ^−1^; probability of symptomatic infection (*σ*) = 0.25; transmissibility (*β*) is calculated per value of *R*_0_

Figure 6 presents the results of stochastic simulations of the model across various population sizes. Comparing the observed symptomatic cases in the no vaccine and aP vaccine eras, we see results qualitatively similar to empirical data [44]: introduction of aP vaccine leads to a higher proportion of *B. pertussis*-free weeks across population sizes (Kolmogorov-Smirnov [KS] test, D = 0.46, p =1.2·10^−9^; panel **a**). However, examination of the asymptomatic population (the true burden) reveals many fewer weeks with no cases of *B. pertussis* (KS test, D = 0.53, p =1.2·10^−12^; panel **b**), which is in fact not different from the pre-vaccine era (KS test, D = 0.07, p = 0.97; panel **c**).

**Fig. 7:**
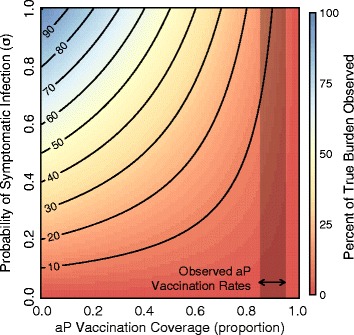
How does an inefficient vaccine affect situational awareness? Figure shows the percent difference in observed infections (symptomatic) from true infections (symptomatic + asymptomatic) at steady state as aP vaccination rate increases and the probability of symptomatic infection increases. Shaded area indicates a range of reasonable aP vaccination rates. At current aP vaccination coverage levels, the majority of cases are asymptomatic and therefore undetected. See Additional file 1 for model details. Parameters: birth rate (*μ*) = death rate (*ν*) = 1/75 years ^−1^; recovery rates for symptomatic (*γ*_*s*_) and asymptomatic (*γ*_*a*_) = 14 days ^−1^; baseline *wP* vaccination rate = 0.9; transmissibility (*β*) is calculated such that *R*_0_=18. Note that previously published values of *R*_0_ for pertussis range from 16–20 [71] to closer to 5 in some populations [72]

Figure 7 demonstrates the percentage of the true infections observed at steady state ([Observed Incidence/Total Incidence-1]*100) as aP vaccination rate increases and the probability of symptomatic infection (*σ*) increases. We find that for realistic aP coverage rates (between 85 % and 95 %), the percentage of total cases expected to be observed is low (<15 *%*), and is highly dependent on the probability of an infection becoming symptomatic (a parameter that is generally not known). These results are likely to be conservative given the low, but unknown, diagnosis rate of asymptomatic infections and known underreporting of symptomatic infections in adults [45].

### Consequences of asymptomatic transmission: increased *B. pertussis* incidence

**Fig. 8:**
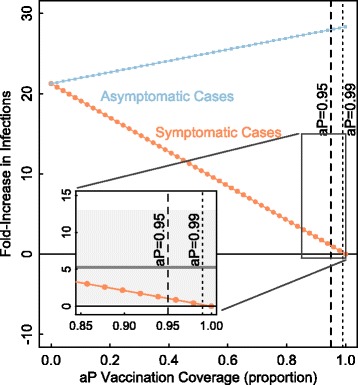
Can an inefficient vaccine lead to increased transmission? Figure demonstrates the fold increase in observed symptomatic and unobserved asymptomatic infections after transitioning from a wP to an aP vaccine. This is calculated by dividing the number of symptomatic or asymptomatic cases with various levels of aP coverage (reported on the x-axis) and 0 % wP coverage by the number of cases with 90 % wP coverage and 0 % aP coverage. This was designed to simulate the switch from wP to aP in the US and UK (going from high wP coverage to coverage with aP). We see an increase in symptomatic cases across a large range of aP vaccination coverage levels. See Additional file 1 for model details. The gray band indicates the empirical 5.4-fold (95 % bootstrap confidence interval: 0.4–13.3) increase in cases in the US comparing 2012 to the years 1985 through 1995. The model recreates the observed increase in cases. Parameters: birth rate (*μ*) = death rate (*ν*) = 1/75 years ^−1^; recovery rates for symptomatic (*γ*_*s*_) and asymptomatic (*γ*_*a*_) = 14 days ^−1^; probability of symptomatic infection (*σ*) = 0.25; baseline *wP* vaccination rate = 0.9; transmissibility (*β*) is calculated such that *R*_0_=18

Figure 8 illustrates the fold increase in observed symptomatic and unobserved asymptomatic infections after transitioning from a wP to an aP vaccine at equilibrium. This is calculated by dividing the number of symptomatic or asymptomatic cases with various levels of aP coverage (reported on the x-axis) and 0 % wP coverage by the number of cases with 90 % wP coverage and 0 % aP coverage. This is done to simulate the switch from wP to aP in the US and UK (going from high wP coverage to coverage with aP), and indicates that a change in vaccine could partially account for the rise in cases.

As aP vaccination coverage increases, asymptomatic infections increase up to nearly 30-fold. We see a substantial increase in the observed numbers of symptomatic cases after wP vaccination is replaced by aP vaccination. At low to moderate levels of aP vaccination, there is a 5- to 15-fold increase in symptomatic cases. Only at extremely high levels of aP vaccination (>99 *%*) is there no change in symptomatic infections. This is in line with the observed rise in *B. pertussis* incidence: cases in 2012 were 5.4-fold (95 % bootstrap confidence interval: 0.4–13.3) higher than cases in years 1985 through 1995 ([46]). This result is similar to previous findings by van Boven et al. (2005) [47], who found that as wP vaccination coverage increased, primary infections (symptomatic) decreased, while secondary infections (subclinical, or asymptomatic) increased.

### Effects of waning immunity to *B. pertussis*

**Fig. 9:**
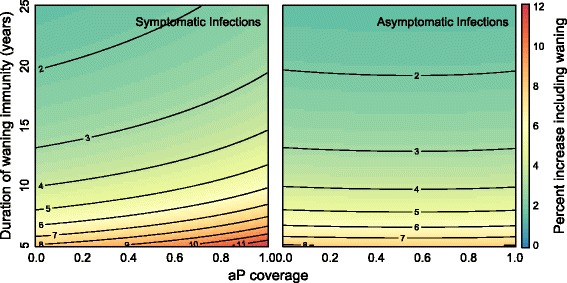
Effects of including waning immunity on symptomatic and asymptomatic infections. Figure shows percent increases in symptomatic and asymptomatic cases at equilibrium after the switch to aP vaccination with inclusion of waning immunity. Parameters: birth rate (*μ*) = death rate (*ν*) = 1/75 years ^−1^; recovery rates for symptomatic (*γ*_*s*_) and asymptomatic (*γ*_*a*_) = 14 days ^−1^; probability of symptomatic infection (*σ*) = 0.25; baseline *wP* vaccination rate = 0.9; transmissibility (*β*) is calculated such that *R*_0_=18

It is clear that waning immunity plays a role in the epidemiology of *B. pertussis*, though estimates of the duration of protection to *B. pertussis* are highly varied [7, 15, 48]. Because the exclusion of waning immunity was a conservative modeling assumption, we focused our analysis on a model where immunity due to vaccination was lifelong. However, as Fig. 9 demonstrates, the inclusion of waning immunity increases symptomatic infections up to 12 % and asymptomatic infections up to 8 %. Importantly, waning immunity would not explain the failure of infant cocooning strategies, the synchrony in age-specific attack rates after the switch to the aP vaccine as presented above, or the observed *B. pertussis* genomic patterns.

## Discussion

In this paper, we have presented empirical evidence — from both case and genomic data — for asymptomatic *B. pertussis* transmission following the switch from the wP to the aP vaccine in the US and UK. Then, using mathematical and computational transmission models, we have demonstrated that an aP vaccine which blocks symptomatic disease but not asymptomatic transmission is able to account for the observed increase in *B. pertussis* incidence; complicates situational awareness surrounding levels of current *B. pertussis* transmission; and potentially biases estimates of vaccine efficacy obtained from case data. When coupled with the laboratory results on asymptomatic transmission in non-human primates from Warfel et al. (2014) and evidence from cross-sectional, human studies in China by Zhang et al. (2014) [12], we conclude that asymptomatic transmission from aP vaccinated individuals to fully susceptible individuals provides the most parsimonious explanation for the observed resurgence of *B. pertussis* in the US and UK, the changes in age-specific attack rates, the observed increase in *B. pertussis* genetic variation, and the multiply demonstrated failure of cocooning unvaccinated infants [35, 36].

Our observation of increased cases due to asymptomatic or subclinical infections has been noted in previous studies of wP vaccination [47–49], as well as examinations of the effects of natural and vaccine-induced protection on the duration of immunity [4, 50]. While these previous studies suggest that asymptomatic infections may account for an increase in whooping cough incidence, they do not provide strong evidence of asymptomatic transmission and do not discuss the failure of infant cocooning. Also, these studies were focused on cases occurring during the wP vaccine era.

There are several limitations to the data presented in this study. First and foremost is a lack of publicly available data to more fully explore the hypotheses suggested. This lack of available data is well known among researchers studying *B. pertussis* [51]. Longer time series of age-stratified data (specifically in infants too young to be vaccinated) would be required to fully explore how the natural periodicity of *B. pertussis* incidence shifted as wP coverage changed. However, these data either do not exist or are unavailable to researchers. A second issue is that clearly not enough time has elapsed since the switch to aP to draw definitive conclusions about the resumption of cycles of *B. pertussis* incidence. While the data appear most consistent with asymptomatic transmission from aP vaccinated individuals, it may be many years before enough time has elapsed to be able to rule out this hypothesis.

In this study we focused on the United States and the United Kingdom where *B. pertussis* incidence has increased in the past 20 years. We acknowledge that this resurgence is not universal and that countries vary greatly in reporting rates, vaccine composition or schedule, historical and current vaccination coverage levels, and the genetic diversity of *B. pertussis* strains [1]. The initial increase in US *B. pertussis* cases also began before the aP switch. However, the empirical evidence we presented, specifically the phylodynamic results, wavelet analysis, changing attack rates, and failure of cocooning, all occurred after the aP switch. It may also be the case that a large factor in the observed resurgence is the improvement in *B. pertussis* diagnostics; however, this would not explain the bulk of the empirical evidence presented here. Disentangling the effects of this variation, in order to evaluate hypotheses to explain the resurgence, will require high resolution case and genetic data. The US and UK have the largest, publicly available, sufficiently granular data, but only the US had a sufficient number of *B. pertussis* genomes available to conduct phylodynamic analyses.

Although the phylodynamic results from the US provide support for an increase in the asymptomatic population size after the switch to the aP vaccine, that analysis has a number of important caveats. First, genomic data were only available through 2005, leaving nearly the last decade without samples. Second, although the posterior median estimate of the sampling rate decreases drastically after the switch to the aP vaccine, the 95 % highest posterior densities overlap. As a result, we are unable to statistically conclude that sampling rates are lower. Third, an increase in underreporting could also explain a decrease in the estimated sampling rate; however, due to changes in *B. pertussis* diagnostics [1, 42] it is unlikely that there has been a substantial *decrease* in underreporting coinciding with the aP switch. Nevertheless, the well-documented issues associated with underreporting of *B. pertussis* cases in adults remains an important area for future phylodynamic studies [52]. Lastly, data availability prevented us from performing phylodynamic analyses in different countries. For example, predictions could be tested for those countries where: a resurgence is also occurring (such as the UK) [1], case counts remain fairly constant and low (such as Italy) [1], molecular evidence highlights the importance of vaccine escape (such as Australia) [30, 53], and where the wP vaccine is still in use (such as Kenya) [54].

As is the case with all models, the one used in this study makes a number of simplifying assumptions. However, most of these assumptions render our conclusions conservative. We assume that wP vaccination is 100 % effective, which is not the case [55]. Relaxing this assumption is analogous to having lower coverage overall, and thus our estimates of fold increase after the aP switch are conservative. Our model does not explicitly account for evolution of the *B. pertussis* bacterium [7, 56] — a factor which may play a large role in the epidemiological dynamics of *B. pertussis*. For example, it has been posited that *B. pertussis* has adapted to vaccination in several European countries. Mooi et al. (2001) identified genetic changes between pre- and post-vaccination strains of *B. pertussis* [8]. Despite this evidence, including evolution would merely increase the number of individuals susceptible to both symptomatic and asymptomatic infection and would yield exactly the opposite pattern of population genomic variation than seen empirically.

Our model also assumes that symptomatic and asymptomatic infections have the same basic reproduction number. Asymptomatic or subclinical/misdiagnosed individuals may spread *B. pertussis* through direct contact, breathing, or coughing [57]. Although coughing may increase transmission, the total bacterial load in the nasopharynx of *B. pertussis*-infected non-human primates is similar between symptomatic and asymptomatic individuals (see Figure one, panel a in [9]). The same study suggested that the duration of higher bacterial loads may be longer in asymptomatic individuals, and that there may not be differences in routes of transmission between asymptomatic and symptomatic individuals. However, and perhaps more importantly, being asymptomatic suggests that individuals may not alter their behavior and thus contact more individuals than a symptomatic individual [58]. Therefore, it seems equally plausible to conclude that the *R*_0_ for aP vaccinated individuals is higher [47]. Future studies should make estimating the distribution of effective reproductive numbers for symptomatic and asymptomatic individuals a priority.

It is important to note that distinguishing true asymptomatic infections from attenuated, symptomatic infections, especially in older age groups, is challenging. If infections were truly asymptomatic, interventions based on teaching clinicians about the potential range of clinical presentations of *B. pertussis* infection would not work, while proper identification of attenuated symptomatic infections could allow for proper increased use of prophylactic measures. Finally, our study assumes a constant probability of symptomatic infection, whereas previous work has shown the probability of becoming symptomatic may depend on the history of exposures of an individual [59]. Future work should explore the probability of symptomatic infection and its potential changes over the life of an individual.

## Conclusions

That there has been a rise in whooping cough incidence in many countries around the globe is irrefutable. The findings presented in Warfel et al., in conjunction with ours, have profound implications for the understanding of *B. pertussis* transmission dynamics and for vaccination policy. Specifically, our results would explain the negative outcomes found in recent studies of postnatal cocooning [35, 36] and would further complicate efforts to achieve herd immunity and possible eradication [60]. Long-term solutions to *B. pertussis* vaccination are necessary, and new vaccines are in development [61, 62]. In the years before a new vaccine is ready for clinical use, other options are necessary for reducing incidence, including vaccination of pregnant women [63, 64] or potentially a switch back to wP vaccination as a priming dose [65–67].

Clearly, more research is necessary, but if our results hold, public health authorities may be facing a situation similar to that of polio, where vaccinated individuals can still transmit infection [68]. This suggests further modifications of recommendations to clinicians for protecting unvaccinated children [69] and ensuring that aP coverage remains high. Our results on the potential surveillance bias associated with *B. pertussis* incidence highlight a critical need for population-wide serological surveys to detect recent infection, studies to examine the genetic diversity of the *B. pertussis* bacterium, more detailed studies of the incidence rate in unvaccinated individuals, and increased active surveillance of attenuated symptomatic *B. pertussis* infections. In light of current evidence and our results, we cannot dismiss the potential far-reaching epidemiological consequences of asymptomatic transmission of *B. pertussis* and an ineffective *B. pertussis* vaccine.

## Additional file

Additional file 1
**Supplementary Information.** Supplementary Information includes model equation and further details, as well as analytical calculations of the steady-state equilibria and *R*_0_. It also includes sensitivity analyses for the force of infection (*β*) and the fraction of symptomatic infection (*σ*).

## References

[1] Jackson DW, Rohani P (2013). Perplexities of pertussis: recent global epidemiological trends and their potential causes. Epidemiol Infect.

[2] CDC Pertussis (Whooping Cough) Surveillance & Reporting. http://www.cdc.gov/pertussis/surv-reporting.html.

[3] Office for National Statistics, UK, Datasets and reference tables. http://www.ons.gov.uk/ons/datasets-and-tables/index.html.

[4] Águas R, Gonçalves G, Gomes MGM (2006). Pertussis: increasing disease as a consequence of reducing transmission. Lancet Infect Dis..

[5] World Health Organization. Progress Towards Global Immunization Goals - 2012. http://www.unicef.org/immunization/files/SlidesGlobalImmunization.pdf.

[6] Gambhir M, Clark TA, Cauchemez S, Tartof SY, Swerdlow DL, Ferguson NM (2015). A Change in Vaccine Efficacy and Duration of Protection Explains Recent Rises in Pertussis Incidence in the United States. PLoS Comput Biol.

[7] Wearing HJ, Rohani P (2009). Estimating the duration of pertussis immunity using epidemiological signatures. PLoS Pathog..

[8] Mooi FR, Van Loo I, King AJ (2001). Adaptation of Bordetella pertussis to vaccination: a cause for its reemergence?. Emerg Infect Dis..

[9] Warfel JM, Zimmerman LI, Merkel TJ (2014). Acellular pertussis vaccines protect against disease but fail to prevent infection and transmission in a nonhuman primate model. Proc Natl Acad Sci..

[10] Storsaeter J, Hallander H, Farrington CP, Olin P, Möllby R, Miller E (1990). Secondary analyses of the efficacy of two acellular pertussis vaccines evaluated in a Swedish phase III trial. Vaccine.

[11] Von Linstow ML, Pontoppidan PL, Cherry JD, Hogh B, von König C-HW (2010). Evidence of Bordetella pertussis infection in vaccinated 1-year-old Danish children. Eur J Pediatr..

[12] Zhang Q, Yin Z, Li Y, Luo H, Shao Z, Gao Y (2014). Prevalence of asymptomatic Bordetella pertussis and Bordetella parapertussis infections among school children in China as determined by pooled real-time PCR: A cross-sectional study. Scand J Infect Dis..

[13] de Melker HE, Versteegh FG, Schellekens JF, Teunis PF, Kretzschmar M (2006). The incidence of *Bordetella pertussis* infections estimated in the population from a combination of serological surveys. J Infect..

[14] Cortese MM, Baughman AL, Brown K, Srivastava P (2007). A “new age” in pertussis prevention: new opportunities through adult vaccination. Am J Prev Med..

[15] Domenech de Cellès M, Riolo MA, Magpantay FMG, Rohani P, King AA (2014). Epidemiological evidence for herd immunity induced by acellular pertussis vaccines. Proc Natl Acad Sci USA.

[16] Warfel JM, Merkel TJ. Reply to Domenech de Cellès et al.: infection and transmission of pertussis in the baboon model. Proc Natl Acad Sci.; 111:718. 10.1073/pnas.1324074111 PMC3932914 24693544

[17] CDC Pertussis Surveillance & Reporting. http://www.cdc.gov/pertussis/surv-reporting.html.

[18] Public Health England, Whooping cough (pertussis) statistics. https://www.gov.uk/government/publications/whooping-cough-pertussis-statistics.

[19] Office for National Statistics, UK, Datasets and reference tables. http://www.ons.gov.uk/ons/datasets-and-tables/index.html.

[20] Project Tycho. http://www.tycho.pitt.edu. copyright 2013, and it was last accessed June 2nd, 2014.

[21] van Panhuis WG, Grefenstette J, Jung SY, Chok NS, Cross A, Eng H (2013). Contagious diseases in the United States from 1888 to the present. N Engl J Med..

[22] Hethcote HW (1997). An age-structured model for pertussis transmission. Math Biosci..

[23] Keeling MJ, Rohani P (2008). Modeling infectious diseases in humans and animals.

[24] Anderson RM, May RM (1992). Infectious diseases of humans: dynamics and control.

[25] Edwards KM (2014). Unraveling the challenges of pertussis. Proc Natl Acad Sci..

[26] Althouse BM, Bergstrom TC, Bergstrom CT (2010). A public choice framework for controlling transmissible and evolving diseases. Proc Natl Acad Sci U S A.

[27] Tanaka MM, Althouse BM, Bergstrom CT (2014). Timing of antimicrobial use influences the evolution of antimicrobial resistance during disease epidemics. Evol Med Publ Health.

[28] Gillespie DT (1977). Exact stochastic simulation of coupled chemical reactions. J Phys Chem.

[29] Chatterjee A, Vlachos DG, Katsoulakis MA (2005). Binomial distribution based tau-leap accelerated stochastic simulation. J Chem Phys..

[30] Bart MJ, Harris SR, Advani A, Arakawa Y, Bottero D, Bouchez V (2014). Global population structure and evolution of Bordetella pertussis and their relationship with vaccination. mBio.

[31] Bouckaert R, Heled J, Kühnert D, Vaughan T, Wu CH, Xie D (2014). BEAST 2: a software platform for Bayesian evolutionary analysis. PLoS Comput Biol..

[32] Darriba D, Taboada GL, Doallo R, Posada D (2012). jModelTest 2: more models, new heuristics and parallel computing. Nat Methods.

[33] Stadler T, Kühnert D, Bonhoeffer S, Drummond AJ (2013). Birth–death skyline plot reveals temporal changes of epidemic spread in HIV and hepatitis C virus (HCV). Proc Natl Acad Sci..

[34] Drummond AJ, Ho SY, Phillips MJ, Rambaut A (2006). Relaxed phylogenetics and dating with confidence. PLoS Biol..

[35] Castagnini LA, Healy CM, Rench MA, Wootton SH, Munoz FM, Baker CJ (2012). Impact of maternal postpartum tetanus and diphtheria toxoids and acellular pertussis immunization on infant pertussis infection. Clin Infect Dis..

[36] Healy CM, Rench MA, Wootton SH, Castagnini LA (2015). Evaluation of the impact of a pertussis cocooning program on infant pertussis infection. Pediatr Infect Dis J..

[37] Miller E, Gay N (1996). Epidemiological determinants of pertussis. Dev Biol Stand..

[38] Gay NJ, Miller E (2000). Pertussis transmission in England and Wales. The Lancet.

[39] Rohani P, Earn DJ, Grenfell BT (2000). Reply to Pertussis transmission in England and Wales. The Lancet.

[40] for Disease Control C (1997). Prevention: Case definitions for infectious conditions under public health surveillance. MMWR.

[41] Shakib J, Wyman L, Gesteland P, Staes C, Bennion D, Byington C (2009). Should the pertussis case definition for public health reporting be refined?. J Public Health Manag Pract..

[42] Cherry JD (2012). Epidemic pertussis in 2012—the resurgence of a vaccine-preventable disease. N Engl J Med..

[43] Bartlett M (1960). The critical community size for measles in the United States. J R Stat Soc Ser A (General).

[44] Rohani P, Earn DJ, Grenfell BT (2000). Impact of immunisation on pertussis transmission in England and Wales. The Lancet.

[45] Rendi-Wagner P, Tobias J, Moerman L, Goren S, Bassal R, Green M (2010). The seroepidemiology of Bordetella pertussis in Israel—estimate of incidence of infection. Vaccine.

[46] CDC Pertussis (Whooping Cough) Surveillance & Reporting. http://www.cdc.gov/pertussis/surv-reporting.html.

[47] van Boven M, Mooi FR, Schellekens JF, de Melker HE, Kretzschmar M (2005). Pathogen adaptation under imperfect vaccination: implications for pertussis. Proc R Soc B Biol Sci..

[48] van Boven M, de Melker HE, Schellekens JF, Kretzschmar M (2000). Waning immunity and sub-clinical infection in an epidemic model: implications for pertussis in the Netherlands. Math Biosci..

[49] Wendelboe AM, Van Rie A, Salmaso S, Englund JA (2005). Duration of immunity against pertussis after natural infection or vaccination. Pediatr Infect Dis J..

[50] Lavine JS, King AA, Bjørnstad ON (2011). Natural immune boosting in pertussis dynamics and the potential for long-term vaccine failure. Proc Natl Acad Sci..

[51] Goldwyn EE, Rohani P (2013). Bias in pertussis incidence data and its implications for public health epidemiology. J Public Health Manag Pract..

[52] Güriş D, Strebel PM, Bardenheier B, Brennan M, Tachdjian R, Finch E (1999). Changing epidemiology of pertussis in the United States: increasing reported incidence among adolescents and adults, 1990-1996. Clin Infect Dis..

[53] Lam C, Octavia S, Ricafort L, Sintchenko V, Gilbert GL, Wood N (2014). Rapid increase in pertactin-deficient Bordetella pertussis isolates, Australia. Emerg Infect Dis..

[54] Mutua MK, Kimani-Murage E, Ettarh RR (2011). Childhood vaccination in informal urban settlements in Nairobi, Kenya: who gets vaccinated?. BMC Public Health.

[55] Bentsi-Enchill AD, Halperin SA, Scott J, MacIsaac K, Duclos P (1997). Estimates of the effectiveness of a whole-cell pertussis vaccine from an outbreak in an immunized population. Vaccine.

[56] Queenan AM, Cassiday PK, Evangelista A (2013). Pertactin-negative variants of Bordetella pertussis in the United States. N Engl J Med..

[57] Warfel JM, Beren J, Merkel TJ (2012). Airborne transmission of Bordetella pertussis. J Infect Dis..

[58] Althouse BM, Hébert-Dufresne L (2014). Epidemic cycles driven by host behaviour. J R Soc Interface.

[59] De Graaf W, Kretzschmar M, Teunis P, Diekmann O (2014). A two-phase within-host model for immune response and its application to serological profiles of pertussis. Epidemics.

[60] Riolo MA, King AA, Rohani P (2013). Can vaccine legacy explain the British pertussis resurgence?. Vaccine.

[61] Locht C, Mielcarek N (2014). Live attenuated vaccines against pertussis. Expert Rev Vaccines.

[62] Meade BD, Plotkin SA, Locht C (2014). Possible options for new pertussis vaccines. J Infect Dis.

[63] Abu Raya B, Srugo I, Kessel A, Peterman M, Bader D, Gonen R (2014). The effect of timing of maternal tetanus, diphtheria, and acellular pertussis (Tdap) immunization during pregnancy on newborn pertussis antibody levels–a prospective study. Vaccine.

[64] Munoz FM, Bond NH, Maccato M, Pinell P, Hammill HA, Swamy GK (2014). Safety and immunogenicity of tetanus diphtheria and acellular pertussis (Tdap) immunization during pregnancy in mothers and infants: a randomized clinical trial. JAMA: J Am Med Assoc.

[65] Sheridan SL, Ware RS, Grimwood K, Lambert SB (2012). Number and order of whole cell pertussis vaccines in infancy and disease protection. JAMA.

[66] Liko J, Robison SG, Cieslak PR (2013). Priming with whole-cell versus acellular pertussis vaccine. N Engl J Med..

[67] Witt MA, Arias L, Katz PH, Truong ET, Witt DJ (2013). Reduced risk of pertussis among persons ever vaccinated with whole cell pertussis vaccine compared to recipients of acellular pertussis vaccines in a large US cohort. Clin Infect Dis.

[68] Fine PE, Carneiro IA (1999). Transmissibility and persistence of oral polio vaccine viruses: implications for the global poliomyelitis eradication initiative. Am J Epidemiol..

[69] Plotkin SA (2013). The pertussis problem. Clin Infect Dis.

[70] Public Health England, Whooping cough (pertussis) statistics. https://www.gov.uk/government/publications/whooping-cough-pertussis-statistics.

[71] Anderson R, May R (1990). Immunisation and herd immunity. Lancet.

[72] Kretzschmar M, Teunis PF, Pebody R. G (2010). Incidence and reproduction numbers of pertussis: estimates from serological and social contact data in five European countries. PLoS Med..

