# Supplementary material for: Asymptomatic transmission and the resurgence of Bordetella pertussis
Source: BMC Med. 2015 Jun 24;13:146. doi: 10.1186/s12916-015-0382-8 (PMC4482312; doi:10.1186/s12916-015-0382-8)
Supplement: Additional file 1 — Supplementary Information. Supplementary Information includes model equation and further details, as well as analytical calculations of the steady-state equilibria and R 0. It also includes sensitivity analyses for the force of infection (β) and the fraction of symptomatic infection (σ). [file 12916_2015_382_MOESM1_ESM.pdf]

Supporting Information for Asymptomatic transmission and the resurgence of Bordetella pertussis

Benjamin M. Althouse, Samuel V. Scarpino

Model Equations

We formulate a deterministic Susceptible, Infected, Removed (SIR) model of *B. pertussis* transmission [1, 2, 3]. Briefly, individuals are born susceptible to *B. pertussis* infection at rate μ, where they are vaccinated with whole-cell (wP) or acellular (aP) *B. pertussis* vaccine at rates wP and aP, respectively. We assume those vaccinated with wP are completely immune to infection, while those vaccinated with aP move into a vaccinated class where they are susceptible to asymptomatic infection. Susceptible individuals become infected with *B. pertussis* at rate β and become symptomatic with probability σ. Individuals recover from symptomatic and asymptomatic infection at rates γ_s and γ_a, respectively. Individuals die at rate ν, which we set equal to μ to keep population size constant. Individuals can wane from protective immunity at rate ω. The equations governing transmission dynamics are:

$$S'(t) = \mu \cdot (1 - wP - aP) - \beta[I_s(t) + I_a(t)]S(t) + \omega R(t) - \nu S(t) \quad (1)$$

$$I_s'(t) = \beta\sigma[I_s(t) + I_a(t)]S(t) - \gamma_s I_s(t) - \nu I_s(t) \quad (2)$$

$$I_a'(t) = \beta(1-\sigma)[I_s(t) + I_a(t)]S(t) + \beta[I_s(t) + I_a(t)]V(t) - \gamma_a I_a(t) - \nu I_a(t) \quad (3)$$

$$V'(t) = \mu \cdot aP - \beta[I_s(t) + I_a(t)]V(t) - \nu V(t) \quad (4)$$

$$R'(t) = \mu \cdot wP + \gamma_s I_s(t) + \gamma_a I_a(t) - \omega R(t) - \nu R(t) \quad (5)$$

We begin simulations with neither wP or aP vaccination. After some time period, t_{wP}, we initiate wP vaccination, and after that at t_{aP}, we stop wP vaccination and begin aP vaccination, similar to replacement of wP by aP vaccines in the mid-1990s [4]. Although this model does not include waning immunity, a process thought to be important for *B. pertussis*, we discuss in the main text how this is a conservative modeling choice with respect to our conclusions.

Stochastic Model

We developed a stochastic version of the model to examine the effects of population size on stochastic fade-outs of *B. pertussis* epidemics. We used a Gillespie stochastic simulation algorithm [5] with the Binomial Tau leap approximation (BTL) [6] with rates and state transition probabilities chosen to match the deterministic model above. BTL was chosen here for efficiency, computational speed and to avoid negative population sizes [6, 7]. Parameters explored were chosen to be identical to those in the main text with an additional death rate of recovered individuals (1/15) to stabilize population sizes. Population sizes were drawn from a power law distribution with scaling exponent -1.2, with a minimum of 10^2 individuals and a maximum of 10^7 individuals.

Steady-state Equilibria

Calculation of the stready-state equilibria of this model is done by equating Equations (1)–(5) to 0 and solving for the state variables, S, I_s, I_a, V, and R. There are two equilibria. The disease-free equilibrium is given by:

$$S^* = \frac{\mu(1 - aP - wP)}{\nu} \quad (6)$$

$$I_s^* = I_a^* = 0 \quad (7)$$

$$V^* = \frac{aP\mu}{\nu} \quad (8)$$

$$R^* = \frac{\mu wP}{\nu}. \quad (9)$$

The other equilibrium has infectious and vaccination classes given by

$$I_s^* = -\frac{\sigma\rho\Big[\beta\mu\big[\sigma\rho(\gamma_a-\gamma_s)+\nu(wP-1)+\gamma_s(wP-1)\big]+\nu(\gamma_a+\nu)(\gamma_s+\nu)\Big]}{\beta(\gamma_s+\nu)\big[\sigma\rho(\gamma_a-\gamma_s)+\nu(wP-1)+\gamma_s(wP-1)\big]} \tag{10}$$

$$I_a^* = \frac{(\sigma\rho-wP+1)\Big[\beta\mu\big[\sigma\rho(\gamma_a-\gamma_s)+\nu(wP-1)+\gamma_s(wP-1)\big]+\nu(\gamma_a+\nu)(\gamma_s+\nu)\Big]}{\beta(\gamma_a+\nu)\big[\sigma\rho(\gamma_a-\gamma_s)+\nu(wP-1)+\gamma_s(wP-1)\big]} \tag{11}$$

$$V^* = -\frac{aP(\gamma_a+\nu)(\gamma_s+\nu)}{\beta\big[\sigma\rho(\gamma_a-\gamma_s)+\nu(wP-1)+\gamma_s(wP-1)\big]} \tag{12}$$

where $\rho = (aP + wP - 1)$ for clarity.

Calculating R_0

To calculate the basic reproduction number, R_0, we follow the formulation as laid out in Diekmann et al. [8]. R_0 is the spectral radius of the Next Generation Matrix, $\mathbf{K}$, (ie: $R_0 = \rho(\mathbf{K}) = \sup\{\mid \lambda \mid : \lambda \in \sigma(\mathbf{K})\}$ where $\sigma(\cdot)$ denotes the spectrum of matrix $\mathbf{K}$). We will decompose $\mathbf{K}$ into two matrices: $\mathbf{T}$, the *transmission matrix*, where $\mathbf{T}_{ij}$ is the rate at which infected individuals in state j infect individuals in state i; and Σ, the *transition matrix*, where Σ_{ij} is the rate an individual in state j transitions to state i. Diekmann et al. show that

$$\mathbf{K} = -\mathbf{E}^T\mathbf{T}\Sigma^{-1}\mathbf{E} \tag{13}$$

Where $\mathbf{T}^{-1}$ is the inverse of matrix $\mathbf{T}$ and $\mathbf{E}$ is a matrix of unit column vectors $\mathbf{e}_{ij}$ for all i such that the ith row of $\mathbf{T}$ is not identically zero.

We start by linearizing the system about the disease free equilibrium. Assuming $\nu = \mu$, Equations (6)-(9) become:

$$S^* = (1 - aP - wP) \tag{14}$$

$$I_s^* = I_a^* = 0 \tag{15}$$

$$V^* = aP \tag{16}$$

$$R^* = wP. \tag{17}$$

Using the model equations given above, we formulate the *infection subsystem* as:

$$\begin{aligned} I_s' &= \beta\sigma[I_s+I_a]S^* - \gamma_s I_s - \nu I_s \\ &= \beta\sigma[I_s+I_a](1-aP-wP) - \gamma_s I_s - \nu I_s \end{aligned} \tag{18}$$

$$\begin{aligned} I_a' &= \beta(1-\sigma)[I_s+I_a]S^* + \beta[I_s+I_a]V^* - \gamma_a I_a - \nu I_a \\ &= \beta(1-\sigma)[I_s+I_a](1-aP-wP) + \beta[I_s+I_a]aP - \gamma_a I_a - \nu I_a \end{aligned} \tag{19}$$

where the (t)s have been dropped for clarity.

The rate of transmission into symptomatic and asymptomatic classes are given by

$$\frac{\partial}{\partial I_a}(I_s') = \beta\sigma(1-aP-wP) \tag{20}$$

$$\frac{\partial}{\partial I_s}(I_a') = \beta(1-\sigma)(1-aP-wP) \tag{21}$$

$$\frac{\partial}{\partial V}(I_a') = \beta aP \tag{22}$$

We find the transmission matrix to be:

$$\mathbf{T} = \begin{pmatrix} \beta\sigma(1-aP-wP) & 0 & 0 \\ 0 & \beta(1-\sigma)(1-aP-wP) & 0 \\ 0 & \beta aP & 0 \end{pmatrix}. \tag{23}$$

Next we calculate the transition matrix, Σ, where the (i, j) entry is the rate at which an individual in state j transitions to state i (excluding infection transitions). Since there are no transitions between infectious states in our infection subsystem, the transition matrix is a diagonal matrix with the entries equal to the demographic and recovery rates of symptomatics, asymptomatics, and aP vaccinated individuals:

$$\Sigma = \begin{pmatrix} -(\nu+\gamma_s) & 0 & 0 \\ 0 & -(\nu+\gamma_a) & 0 \\ 0 & 0 & \mu a P - \nu \end{pmatrix}. \tag{24}$$

This makes finding the inverse of Σ trivial, it is simply the reciprocal of each non-zero entry. Now, since none of the rows of $\mathbf{T}$ are identically zero, $\mathbf{E}$ is simply the 4-by-4 identity matrix and our next generation matrix (NGM), is $\mathbf{K} = -\mathbf{E}^T\mathbf{T}\Sigma^{-1}\mathbf{E} = -\mathbf{T}\Sigma^{-1} =$

$$\begin{pmatrix} -\frac{\beta\sigma(1-aP-wP)}{-(\nu+\gamma_s)} & 0 & 0 \\ 0 & -\frac{\beta(1-\sigma)(1-aP-wP)}{-(\nu+\gamma_a)} & 0 \\ 0 & -\frac{aP\beta}{-(\nu+\gamma_a)} & 0 \end{pmatrix}. \tag{25}$$

The eigenvalues of $\mathbf{K}$ are

$$\left\{0,\ \frac{\beta(\sigma-1)(aP+wP-2)}{\gamma_a+\nu},\ -\frac{\beta\sigma(aP+wP-2)}{\gamma_s+\nu}\right\}. \tag{26}$$

R_0 is defined as the dominant eigenvalue of $\mathbf{K}$, which is determined by the values of the parameters. The second eigenvalue corresponds to the R_0 of the asymptomatic strain, and the third to the R_0 of the symptomatic strain. Thus, R_0 for the entire system is given by the sum:

$$R_0 = \frac{\beta(\sigma-1)(aP+wP-1)}{\gamma_a+\nu} - \frac{\beta\sigma(aP+wP-1)}{\gamma_s+\nu} \tag{27}$$

$$= \frac{(1-aP-wP)\beta(\gamma_s+\nu+\gamma_a\sigma-\gamma_s\sigma)}{(\gamma_a+\nu)(\gamma_s+\nu)} \tag{28}$$

Different Forces of Infection

Asymptomatic infection may be less transmissible than symptomatic infection due to less shedding of bacteria through coughing. On the other hand, symptomatic individuals may have a smaller force of infection due to self isolation. Thus, in the main text we assume equal forces of infection. To assess sensitivity of our results to this assumption, we can formulate the model with unequal forces of infection, β_s and β_a, for symptomatic and asymptotic infections, respectively. We modify the infection term

$$\beta(1-\sigma)[I_s(t)+I_a(t)]S(t) \to (1-\sigma)[\beta_s I_s(t)+\beta_a I_a(t)]S(t) \tag{29}$$

The steady-state equilibrium becomes

$$I_s^* = -\frac{\sigma(aP+wP-2)\big(\beta_a\mu(\gamma_s+\nu)(\sigma(aP+wP-2)-wP+2)-(\gamma_a+\nu)\big(\beta_s\mu\sigma(aP+wP-2)+\nu^2+\gamma_s\nu\big)\big)}{(\gamma_s+\nu)(\beta_a(\gamma_s+\nu)(\sigma(aP+wP-2)-wP+2)-\beta_s\sigma(aP+wP-2)(\gamma_a+\nu))} \tag{30}$$

$$I_a^* = \frac{(\sigma(aP+wP-2)-wP+2)\big(\beta_a\mu(\gamma_s+\nu)(\sigma(aP+wP-2)-wP+2)-(\gamma_a+\nu)\big(\beta_s\mu\sigma(aP+wP-2)+\nu^2+\gamma_s\nu\big)\big)}{(\gamma_a+\nu)(\beta_a(\gamma_s+\nu)(\sigma(aP+wP-2)-wP+2)-\beta_s\sigma(aP+wP-2)(\gamma_a+\nu))} \tag{31}$$

$$V^* = \frac{aP(\gamma_a+\nu)(\gamma_s+\nu)}{\beta_a(\gamma_s+\nu)(\sigma(aP+wP-2)-wP+2)-\beta_s\sigma(aP+wP-2)(\gamma_a+\nu)}. \tag{32}$$

And R_0,

$$R_0 = (1-aP-wP)\left(\frac{\beta_a(\sigma-1)}{(\gamma_a+\nu)} - \frac{\beta_s\sigma}{(\gamma_s+\nu)}\right). \tag{33}$$

For a less transmissible asymptomatic infection, none of the results change qualitatively (Figure S1). As β_a decreases relative to β_s, R_0 decreases modestly (Figure S2).

Sensitivity of Dynamics to Rate of Asymptomatic Infection (σ)

Figure S3 shows the lack of sensitivity of the dynamics to changes in asymptomatic infection rate (σ).

References

[1] Hethcote, H.W.: An age-structured model for pertussis transmission. Mathematical biosciences **145**(2), 89–136 (1997)

[2] Keeling, M.J., Rohani, P.: Modeling Infectious Diseases in Humans and Animals. Princeton University Press, Princeton (2008). http://www.loc.gov/catdir/toc/fy0805/2006939548.html

[3] Anderson, R.M., May, R.M.: Infectious Diseases of Humans: Dynamics and Control vol. 28. Oxford university press, Oxford, UK (1991)

[4] Edwards, K.M.: Unraveling the challenges of pertussis. Proceedings of the National Academy of Sciences **111**(2), 575–576 (2014)

[5] Gillespie, D.: Exact stochastic simulation of coupled chemical-reactions. In: Abstracts of Papers of the American Chemical Society, vol. 173, pp. 128–128. AMER CHEMICAL SOC, 1155 16TH ST, NW, WASHINGTON, DC 20036 (1977)

[6] Chatterjee, A., Vlachos, D.G., Katsoulakis, M.A.: Binomial distribution based tau-leap accelerated stochastic simulation. J Chem Phys **122**(2), 024112 (2005). doi:10.1063/1.1833357

[7] Pineda-Krch, M.: Gillespiessa: Implementing the stochastic simulation algorithm in r. Journal of Statistical Software **25**(12), 1–18 (2008)

[8] Diekmann, O., Heesterbeek, J.A.P., Roberts, M.G.: The construction of next-generation matrices for compartmental epidemic models. J R Soc Interface (2009). doi:10.1098/rsif.2009.0386

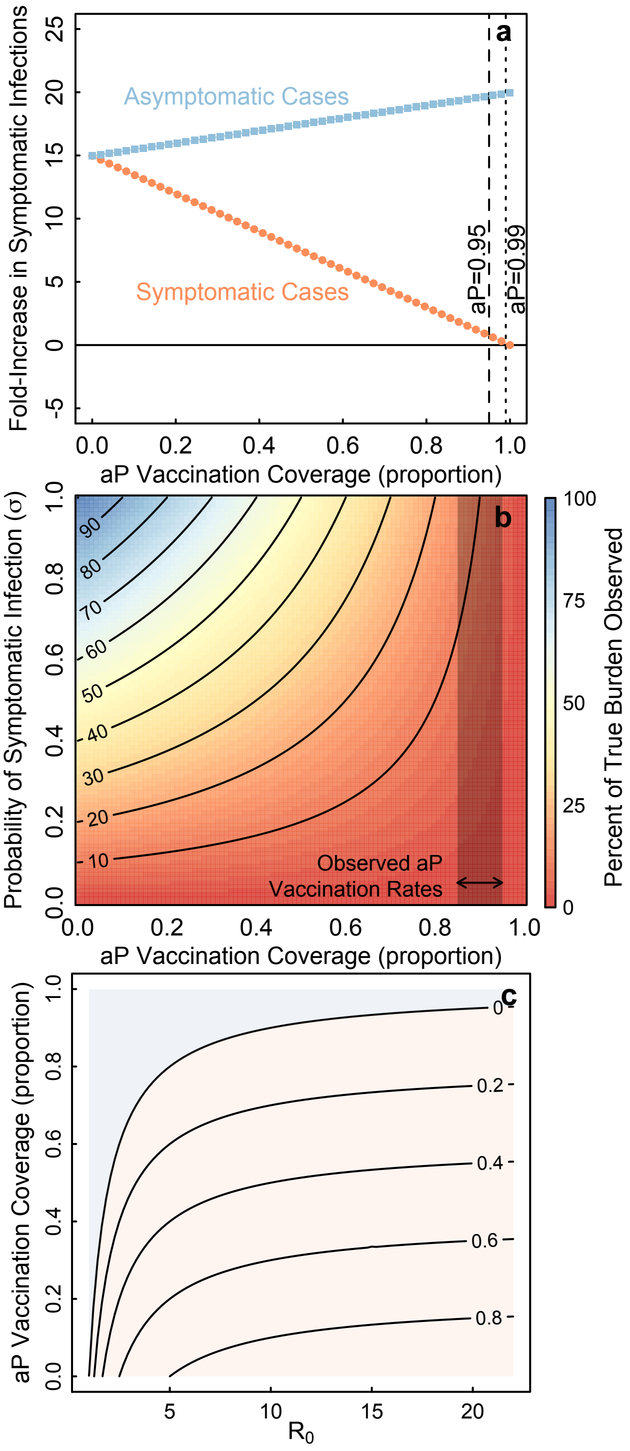

Figure S1: **The effects of ineffective *B. pertussis* vaccination under lower asymptomatic transmission** Figure is analogous to the figures in the main text with $\beta_a = \beta_s/10$. Other parameters: $\mu = \nu = 1/75$ years^{-1}; $\gamma_s = \gamma_a = 14$ days^{-1}; $\sigma = 0.25$; $wP = aP = 0$; $R_0 = 18$.

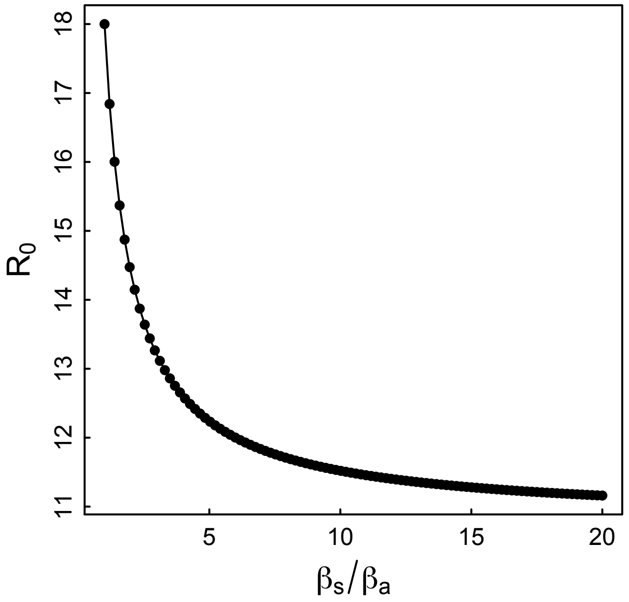

Figure S2: R_0 **changes as asymptomatic infections become less transmissible** Figure shows R_0 decreasing as asymptomatic infections become less transmissible relative to symptomatic infections (β_s/β_a). Parameters: $\mu = \nu = 1/75$ years^{-1}; $\gamma_s = \gamma_a = 14$ days^{-1}; $\sigma = 0.25$; $wP = aP = 0$.

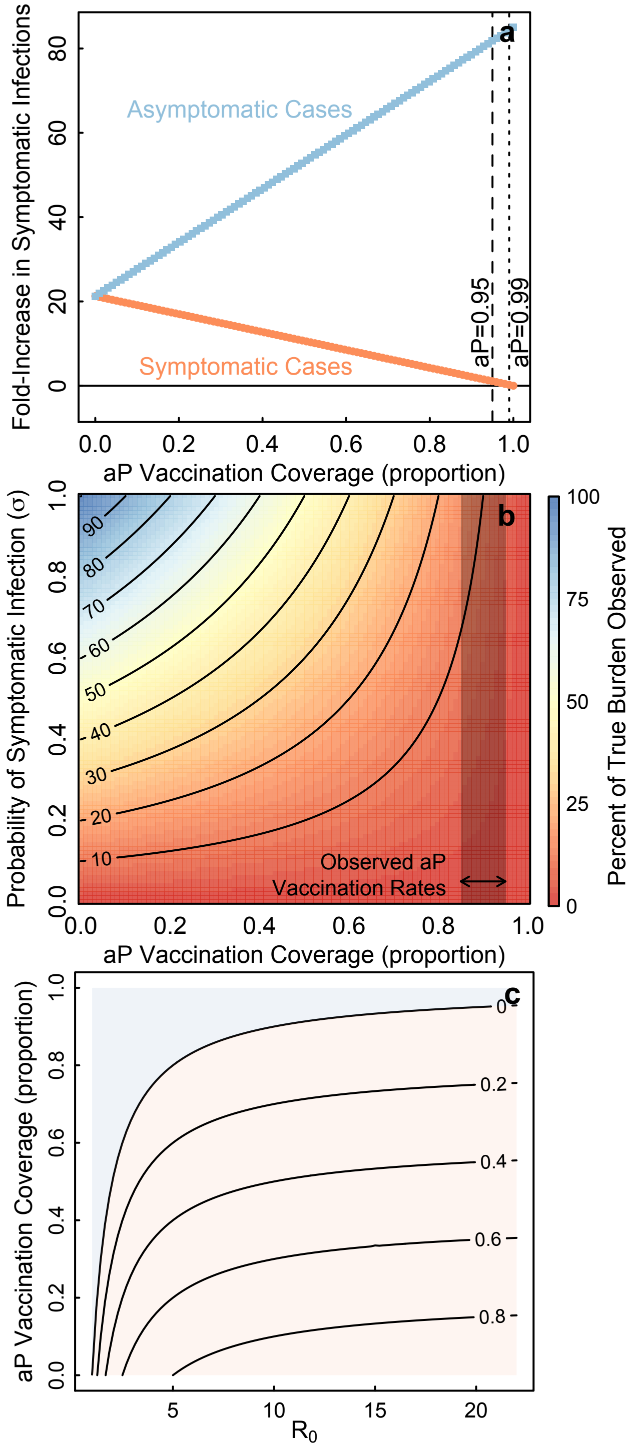

Figure S3: **The effects of ineffective *B. pertussis* vaccination under higher asymptomatic infection rate** Figure is analogous to figures 1, 2, and 5 in the main text with $\sigma = 0.75$. Other parameters: $\mu = \nu = 1/75$ years^{-1}; $\gamma_s = \gamma_a = 14$ days^{-1}; $wP = aP = 0$; $R_0 = 18$.
